# Supplementary material for: Sub-10-fs control of dissociation pathways in the hydrogen molecular ion with a few-pulse attosecond pulse train
Source: Nat Commun. 2016 Sep 20;7:12835. doi: 10.1038/ncomms12835 (PMC5494193; doi:10.1038/ncomms12835)
Supplement: Supplementary Information — Supplementary Figures 1-4 and Supplementary References. [file ncomms12835-s1.pdf]

Supplementary Information

Supplementary Figures

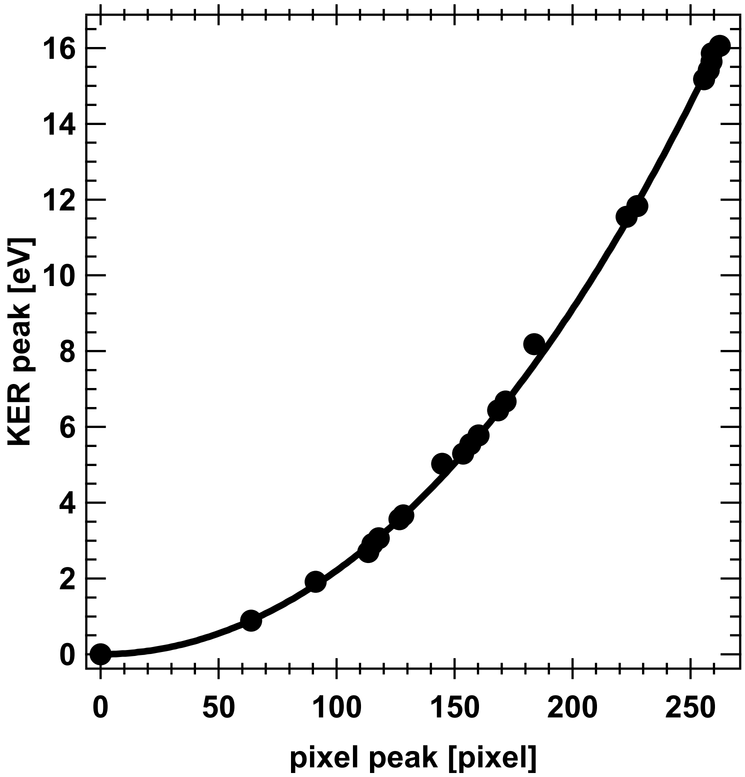

Supplementary Figure 1: Peak positions of the kinetic energy release (KER) versus those of the pixel in sCMOS camera. We have calibrated the KER of H^+ fragments with the manner same as that described in Supplementary Note 3 and shown in Supplementary Figure 4(b) in ref.[38]. First, we calculate the magnitude square of the Fourier transform of the delay-pixel spectrogram for the parallel component of the sliced image of H^+ fragments. We find distinct peaks in the frequency-pixel spectrogram similar to those appearing in Fig. 6 (a) in the main text, although the vertical axis of the spectrogram is proportional to the pixel number of the sCMOS camera, not the KER. Then, we determine which pair of vibrational states generates each peak by identifying each peak frequency as the beat frequency calculated from the known binding energies of the vibrational states. Afterward, we compare the frequency-pixel spectrogram with the FKS in Fig. 6(c) in the main text to determine which peak KER corresponds to each peak pixel. The resultant peak KERs corresponding to peak pixels are plotted as solid circles in Supplementary Figure 1. The peak KER ($K_{\rm pk}$) should be proportional to the square of the peak pixel number ($P_{\rm pk}$). Nevertheless, we added a small quartic correction term to fit the solid circles to reduce χ^2 for the fitting curve, shown as a solid curve in this Supplementary Figure 1, i. e., $K_{\rm pk} = aP_{\rm pk}^2 + bP_{\rm pk}^4$. The coefficients a and b are estimated by χ^2 fitting to this polynomial function, resulting in a =2.192±0.0175×10^{-4} [eV/pixel^2] and b =2.14±0.290×10^{-10} [eV/pixel^4]. χ^2 =0.109 [eV], which is much smaller than the smallest KER peak (∼2 eV) relevant to the dissociation pathway control. Thus, we conclude that the inaccuracy of the KER calibration does not affect the analysis in the main text. Note that the KER resolution per pixel at 16 [eV], at which the highest KER peak emerges (H11 component) in the FKS spectrogram of Fig. 6(a), is 0.13 [eV/pixel]. Under this KER resolution, the gradual increase (∼0.2 [eV/peak]) in the KER peaks from (2,3) to (5,6) in the H11 component of Fig. 6(a) is successfully resolved.

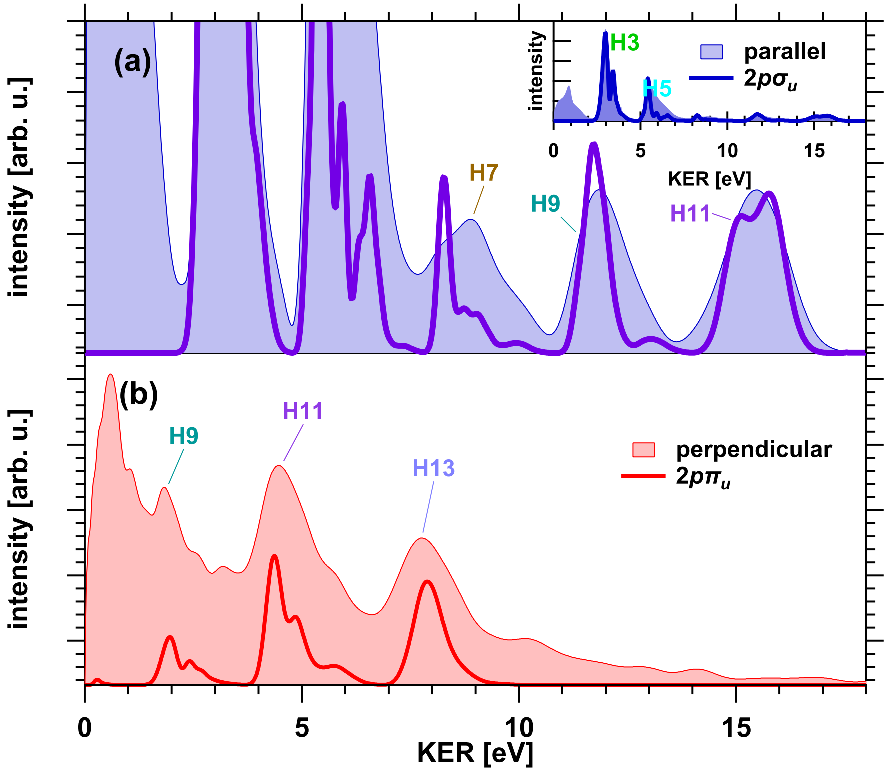

Supplementary Figure 2: Comparison between the theoretical models and experimental results of KER spectra. (a) Magnified view of the KER spectrum of H^+ fragments parallel to the polarization direction of the APT (thin curve with shaded area) in the experiment compared with magnified view of the KER spectrum calculated from our theoretical model assuming that the H^+ fragments are generated from the $2p\sigma_u$ state (thick solid curve). The entire spectra are shown in the inset. Each peak position for each harmonic contribution in the experimental spectrum is in good agreement with that in the calculated spectrum. Fine modulations appearing in the calculated spectral shape are less clearly observed in the experimental spectrum because of the finite spectral resolution of our ion spectrometer. This, however, does not influence our conclusion that the H^+ fragments yielded originate from the $2p\sigma_u$ state. (b) KER spectrum of H^+ fragments perpendicular to the polarization direction of the APT (thin curve with shaded area) in the experiment compared with the KER spectrum calculated from our theoretical model assuming that the H^+ fragments are generated from the $2p\pi_u$ state (thick solid curve). We can see from the experimental spectrum that a broad hump ranging from 0 eV to 15 eV, in addition to each peak corresponding to each harmonic component, emerges as a background signal. This background signal is not reproduced in the calculated spectrum, and thus there may be a contribution from other dissociation processes, such as the autoionization of doubly excited states of the H_2 molecule.

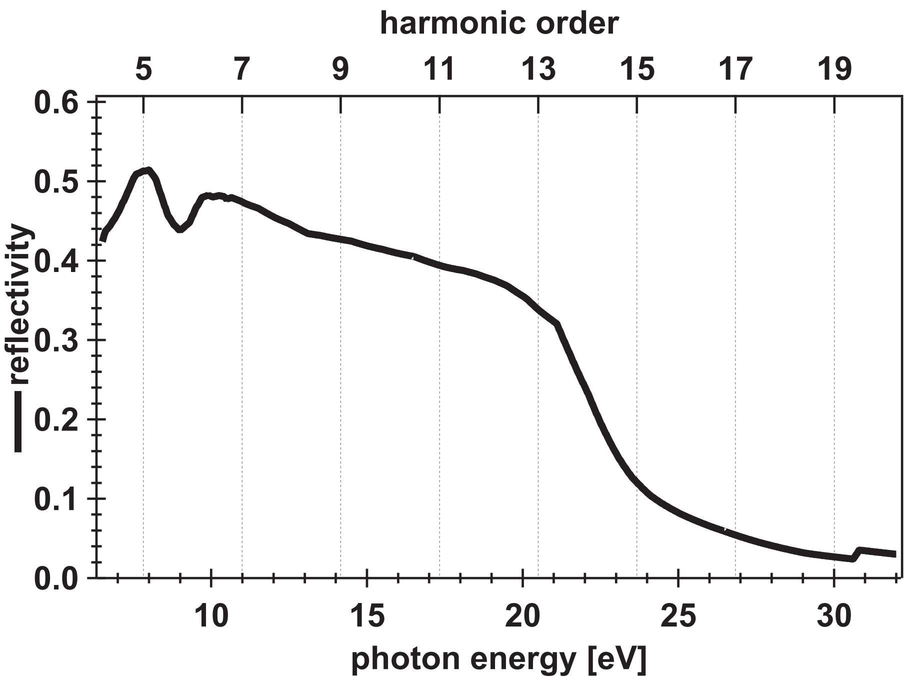

Supplementary Figure 3: Reflectivity of the SiC mirror. This is calculated from the optical constants shown in ref.[1] of this Supplementary Information. The orders of the harmonic components contained in the attoscond pulse train at the peak photon energies are indicated on the top axis. The reflectivity for the 13th- and lower-order harmonic components is expected to be more than 30%. The measured reflectivity for the harmonic components at the Brewster incident angle to the fundamental laser pulse (69°) is reported in ref.[2] of this Supplementary Information.

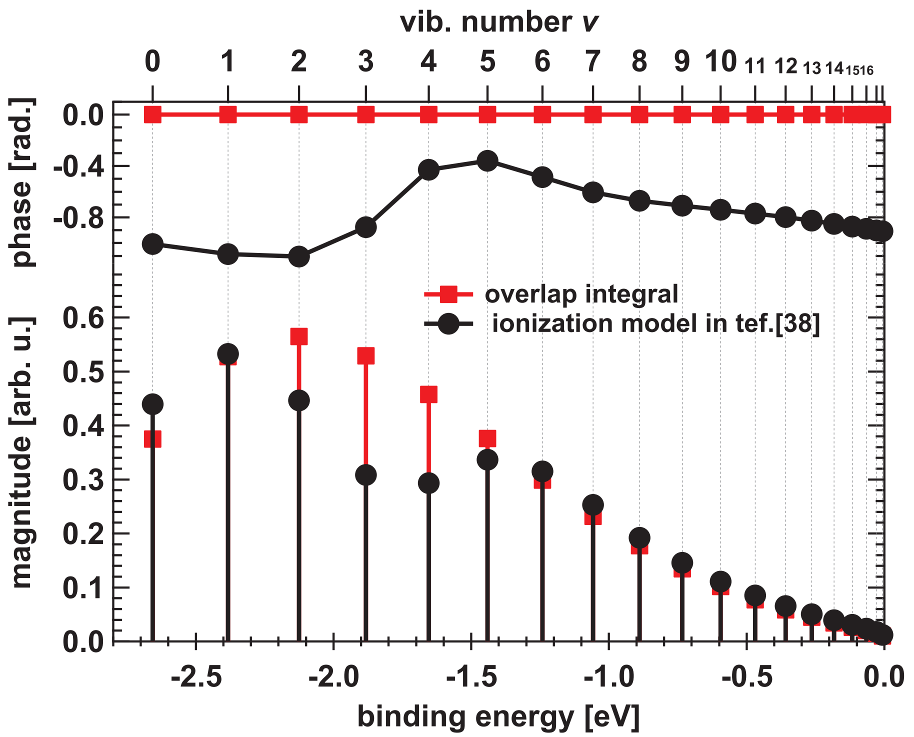

Supplementary Figure 4: Complex amplitude of each vibrational function contained in the wavepacket. We calculate delay-KER spectrograms shown in Figs. 5(c) and (d) in the main text based on the theoretical model described in refs.[37] and [38]. The initial wavepacket in this model is assumed to be $\sum_\nu a_\nu \chi_\nu^g(R)$, where $\chi_\nu^g(R)$ is the νth vibrational wavefunction at the internuclear distance R and a_ν is the complex amplitude of the νth vibrational wavefunction. We show the magnitude of a_ν ($|a_\nu|$) as solid circles with vertical lines and the phase ($\arg\{a_\nu\}$) as solid circles with connecting lines in the lower and upper panels of this Supplementary Figure 4, respectively. The complex amplitudes from $\nu = 0$ to $\nu = 8$ are the same as those depicted as hollow circles in Fig. 4(a) in ref.[38]. The complex amplitudes with $\nu > 8$ were calculated in the manner described in ref.[38]. The magnitudes at $\nu = 2$, 3, and 4 somewhat deviate from those obtained from the overlap integral (depicted as solid squares with vertical lines) between the ground vibrational wavefunction of the neutral H_2 molecule and $\chi_\nu^g(R)$. The phase is also modulated. These features are owing to the fact that the photon energy of the 11th-order harmonic component in the attosecond pulse train is between the ionization energy of the H_2 molecule and the dissociation limit of H_2^+. The model is described in detail in Supplementary Note 5 of ref.[38].

Supplementary References

[1] Palik, E. D., *Handbook of Optical Constants of Solids,* Academic Press, New York (1985).

[2] Kojima, Y., Amani Eilanlou, A., Furukawa, Y., Nabekawa, Y., Takahashi, E. J., Kannari, F. and Midorikawa, K., *Material Survey for a Novel Beam Splitter Separating High-Order Harmonics from High-Average-Power Fundamental Pulses.* Jpn. J. Appl. Phys. **51** (2012) 062601.
